# Decreased Bioelectrical Impedance Phase Angle in Hospitalized Children and Adolescents with Newly Diagnosed Type 1 Diabetes: A Case-Control Study

**DOI:** 10.3390/jcm7120516

**Published:** 2018-12-04

**Authors:** Paweł Więch, Dariusz Bazaliński, Izabela Sałacińska, Monika Binkowska-Bury, Bartosz Korczowski, Artur Mazur, Maria Kózka, Mariusz Dąbrowski

**Affiliations:** 1 Institute of Nursing and Health Sciences, Faculty of Medicine, University of Rzeszów, 35-959 Rzeszów, Poland; darek.bazalinski@wp.pl (D.B.); izabela.salacinska@wp.pl (I.S.); monika.binkowska@yahoo.com (M.B.-B.); mariusz.dabrowski58@gmail.com (M.D.); 2 Pediatric Department, Clinical Provincial Hospital No. 2 in Rzeszów, Faculty of Medicine, University of Rzeszów, 35-301 Rzeszów, Poland; korczowski@op.pl (B.K.); drmazur@poczta.onet.pl (A.M.); 3 Department of Clinical Nursing, Faculty of Health Sciences, Collegium Medicum, Jagiellonian University, 31-501 Kraków, Poland; makozka@cm-uj.krakow.pl; 4 Diabetic Outpatient Clinic, Medical Center “Beta-Med”, 35-073 Rzeszów, Poland; mariusz.dabrowski58@gmail.com

**Keywords:** type 1 diabetes mellitus, bioelectrical impedance analysis, phase angle, children, adolescents

## Abstract

The aim of this study was to assess the body composition and nutritional status of hospitalized pediatric patients with newly diagnosed type 1 diabetes by using bioelectrical impedance analysis (BIA) with phase angle (PA) calculation. PA is considered to be a useful and very sensitive indicator of the nutritional and functional status, and it has not yet been evaluated in such a population. Sixty-three pediatric patients aged 4 to 18 years, with newly diagnosed type 1 diabetes, were included in the study. The control group consisted of 63 healthy children and adolescents strictly matched by gender and age in a 1:1 case: control manner. In both groups, BIA with PA calculation was performed. Diabetic patients, in comparison to control subjects, had a highly significantly lower PA of 4.85 ± 0.86 vs. 5.62 ± 0.81, *p* < 0.001. They also demonstrated a lower percentage of body cell mass (BCM%), 46.89 ± 5.67% vs. 51.40 ± 4.19%, p < 0.001; a lower body cell mass index (BCMI), 6.57 ± 1.80% vs. 7.37 ± 1.72%, *p* = 0.004; and a lower percentage of muscle mass (MM%), 44.61 ± 6.58% vs. 49.40 ± 7.59%, *p* < 0.001, compared to non-diabetic controls. The significantly lower PA value in diabetic patients indicate their worse nutritional and functional status compared to healthy subjects. To assess the predictive and prognostic value of this finding in this population, further prospective studies involving larger sample of patients are required.

## 1. Introduction

Application of bioelectrical impedance analysis (BIA) to assess the body composition dates back to late 1980s [1]. Although differences between results obtained by BIA and Dual-Energy X-Ray Absorptiometry (DXA) are observed both in adult and in pediatric populations, BIA is considered as an easy to use, reliable, safe, non-invasive, and non-expensive method of the assessment of body composition, and it has been widely used in a number of epidemiological studies [2,3,4,5].

Bioelectrical impedance phase angle (PA) is considered to be a more sensitive and more accurate indicator of the nutritional and functional status than BIA alone, because PA better reflects the degree of cellular health and indicates early water shift from intracellular to extracellular compartment in subjects with malnutrition [6,7,8].

The reference values of PA in healthy subjects differ depending on age, sex, and body mass index (BMI) [9,10,11]. The PA values in adults are different compared with children and adolescents. Growing up is associated with increasing phase angles, a situation that is probably due to an increase in cell mass with age, especially in periods of childhood and adolescence [9]. This trend was observed by Redondo-Del-Río et al. for both genders (4–5 years: 5.2 vs. 12–13 years: 5.7–5.8 vs. 15–16 years: 6.1–6.5) [10].

Norman et al. summarized in their review the prognostic value of PA in dialysis subjects, patients with cancer, liver cirrhosis, heart failure, systemic sclerosis, human immunodeficiency virus (HIV) positive, geriatric, and surgical patients. In all cases lower PA values were associated with increased mortality and lower survival rates [12]. PA also correlates with function, disease severity, and prognosis in people with stable chronic obstructive pulmonary disease (COPD) [13]. Low PA at hospital admission appeared also to be associated with malnutrition and nutritional risk [14]. In the study by Zhang et al., patients with a better nutrition state demonstrated relatively higher PA than the patients with severe malnutrition, which suggests that PA has the potential to be a quantitative index in assessing nutrition state, while current assessing methods such as the Subjective Global Assessment (SGA) contain many subjective factors [7]. Thus, PA can be considered not only as an indicator of nutritional status but also as an important prognostic factor of malnutrition risk and clinical outcome, especially in chronic diseases leading to malnutrition and cachexia. The European Society for Clinical Nutrition and Metabolism (ESPEN) strongly recommends PA as a reliable prognostic nutritional measure [15]. Currently, it is expected that nutrition state assessment based on PA will be established and implemented in clinical trials [7].

The primary objective of our study was to assess the PA value in hospitalized children and adolescents with newly diagnosed type 1 diabetes compared to healthy controls. To our knowledge, it is the first study in such a group, as through searching Embase, Web of Science, PubMed, and Scopus databases by use of the terms “type 1 diabetes mellitus” and “children”; combined with “body composition” or/and “phase angle”; “bioelectrical impedance analysis”, “type 1 diabetes mellitus”, and “children”; and combined with “nutritional status” and “phase angle”, we found only two full-text papers assessing the PA in diabetic patients, but both of them were conducted in adult populations [16,17].The search was limited to articles published in English from 1990 to 30 July 2018. The secondary objective was to analyze body composition in this group of patients.

## 2. Experimental Section

### 2.1. Ethics

The study was approved by the institutional Bioethics Committee at the University of Rzeszów (Resolution No. 5/02/2012) and by all appropriate administrative bodies. The study was conducted in accordance with ethical standards laid down in an appropriate version of the Declaration of Helsinki and in Polish national regulations.

### 2.2. Subjects

**Figure 1:**
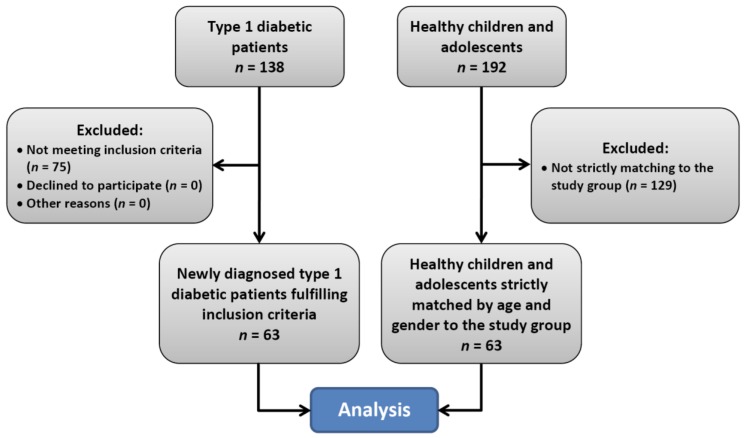
Flow chart of the selection of study participants.

This pair-matched, case control study was carried out between March 2012 and August 2014. Sixty-three children and adolescents (28 girls, 44.4%) with type 1 diabetes hospitalized in the Clinical Department of Pediatrics with the Pediatric Neurology Unit at the Clinical Regional Hospital No. 2 in Rzeszow comprised the study group. The flow chart demonstrating the selection of the study and control groups is presented in Figure 1, while characteristics of both groups are presented in Table 1.

The inclusion criteria were as follows: newly diagnosed type 1 diabetes; age 4 to 18 years; no other autoimmune or chronic diseases having impact on height, weight, or nutritional status; and written informed consent signed by parents or legal guardians and also by adolescents aged over 16 years. The control group consisted of the same number of children and adolescents taken from primary, junior high, and high schools from urban and rural areas. The inclusion criteria for this group were the same, with the exception of having type 1 diabetes. The diabetic and healthy subjects were strictly matched by gender and age (to the nearest possible date of birth) in a 1:1 case:control manner.

### 2.3. Assessments

Body height was measured to the nearest 0.1 cm using a portable stadiometer (Seca 213). The measurements were performed under standard conditions in an upright position and barefoot. Body mass was assessed with an accuracy of 0.1 kg using a personal scale (Seca 799). Body mass index (BMI) was calculated as weight (kg)/height (m)^2^. To avoid bias associated with dehydration at the time of diabetes diagnosis, in the first step, acid–base and water–electrolyte balance in the study group were normalized. In the treatment of hyperglycemia, a continuous intravenous insulin infusion was used in the first several hours after admission. After glucose metabolism control was achieved, children and adolescents were switched to subcutaneous multiple daily injections of insulin using the basal/bolus method. The assessment of body composition and nutritional status in diabetic subjects was performed between the 5th and 7th days after admission after when stable metabolic state was achieved. BIA was performed using an AKERN BIA 101 analyzer (Akern SRL, Pontassieve, Florence, Italy). PA was calculated from the following equation:

(1) $$\mathrm{PA}=(\frac{X_{C}}{R})\times (\frac{180{}^{\circ}}{\pi })\;(\mathrm{Xc}—\mathrm{reactance},\;R—\mathrm{resistance})$$

The equations used by the software to assess the specific parameters are restricted property of the company, but to a significant degree, they are based on computed algorithms developed by Sun S. et al. [19] and Horlick et al. [20]. According to widely accepted and available methodologies [21], participants were in a fasting state and, before measurement (24 h), they did not practice physical exercises. Before the BIA measurement, they were asked to empty the bladder completely and to remove all clothing and metal elements. The measurements were carried out between 07:00 and 12:00 in a fasting state, in the supine position, with abducted upper (30°) and lower (45°) extremities, and after at least 5 min of rest. A tetrapolar system with a contralateral mode was used (amplitude of measured current: 800 uA; sinusoidal; 50 kHz). To ensure reliability and repeatability of the results obtained, two measurements, one after another, were performed. All the measurements were performed by the same person. The accuracy of the equipment was checked before the measurements with a 500 ohm resistor supplied by the manufacturer. The disposable electrodes (Biatrodes, Pontassieve, FI, Italy; the individual impedance of a single electrode: 25–30 Ω; compliance with the Medical Directive 93/42/EEC; accordance with ISO 10993-1:2003) were placed on the dorsal surface of the right upper limb (over the wrist) and the right lower limb (on the ankle) while the minimum distance between the two electrodes was 5 cm. The results were transferred and analyzed by specialized software (Bodygram1_31 by AKERN).

In all children and adolescents, weight and height was measured and BMI was calculated. The obtained results were compared to Polish normative values charts according to age and gender [18]. Weight was divided into five categories according to percentiles: <3 low-to-age weight, 3–10 alert level, 10–90 normal weight, 90–97 alert level, and >97 high-to-age weight. Height was divided into three percentile categories: >97 high-to-age height, 3–97 normal range, and <3 low-to-age height. BMI was divided into four categories: underweight, normal weight, overweight, and obese, according to normative charts for age and gender. BIA analysis included: fat mass (FM), fat free mass (FFM) muscle mass (MM) (kg and %), total body water (TBW), intra- and extracellular water (ICW and ECW; liters and %), body cell mass (BCM; kg and %), and body cell mass index (BCMI). Upon resistance and reactance results, phase angle was calculated. In addition, fat mass index (FMI) and fat free mass index (FFMI) were calculated.

Phase angle results were compared between diabetic and control groups as a whole and separately for each gender. In addition, we analyzed PA values in relation to the reference values for age, gender, and BMI obtained by Bosy-Westphal et al. in the study conducted in Germany, the neighboring country, which can be considered applicable for our population [9].

### 2.4. Statistical Analysis

Statistical analysis of the data was performed using SigmaPlot for Windows, version 12.5 (Systat Software Inc., San Jose, CA, USA). The continuous data are presented as mean ± SD (standard deviation). Differences between study and control groups were analyzed using a two-tailed Student’s t-test for independent samples after performing a Shapiro-Wilk normality test and a constant variance test. In the case of normality and constant variance test failure, the Mann-Whitney rank sum test was performed. The categorical data were compared using the *χ*2 test. The linear correlation between PA and HbA1c was assessed using the Pearson product moment correlation test after checking the normality of distribution by the Shapiro-Wilk test. A *p*-value of <0.05 was considered statistically significant.

## 3. Results

The phase angle value in diabetic patients was highly significantly lower compared to healthy subjects. Among components of the PA equation, only reactance appeared to be significantly different between the study and control groups (Table 2) regardless of gender (Table 3). Resistance, although higher in diabetic subjects, did not reach statistical significance. Although the muscle mass and body cell mass measured in kg were not significantly different between the diabetic and control groups, MM%, BCM%, and BCMI were also significantly lower in diabetic subjects (Table 2). The differences remained significant when these variables were also analyzed separately for each gender (Table 3). 

We also found significant differences between the study and control group for both genders in the analysis of PA values in relation to the reference values for a healthy population obtained by Bosy-Westphal et al. in the study conducted in Germany, the neighboring country [9]. In our study, among girls with diabetes, only 14 (50.0%) had a PA value equal or higher than the 10th percentile expected for gender, age and BMI, while among healthy girls such number was 27 (96.4%), *p* < 0.001 for the difference. Among boys these numbers were 20 (57.1%) and 30 (85.7%) respectively and this difference was also statistically significant, *p* = 0.017. The more detailed results are presented in the Table 4.

The mean HbA1c value in children and adolescents with newly diagnosed type 1 diabetes was 11.37% ± 2.26% (100.7 ± 24.7 mmol/mol). Girls had a mean HbA1c value significantly higher compared to boys, 12.22% ± 1.89% (110.1 ± 20.7 mmol/mol) vs. 10.71% ± 2.32% (93.4 ± 25.3 mmol/mol), respectively, *p* = 0.012. A trend towards negative linear correlation between the PA value and the HbA1c value in diabetic patients was observe; however, it did not attain a statistical significance (*r* = −0.220, *p* = 0.085).

In a subgroup of patients (four with newly diagnosed diabetes and nine with longer-lasting diabetes), we performed a follow-up after 13 to 21 (mean of 15.5) months of treatment. We found a significant improvement in the mean PA value and BCMI (from 5.52 ± 0.77 to 5.84 ± 0.67, *p* = 0.003, and from 6.85 ± 1.99 to 8.04 ± 2.15, *p* < 0.001, respectively). However, at baseline, six patients had a PA value above the mean value expected for age, gender, and BMI, while at follow-up, this number decreased to five. The percentage of FM, FFM, TBW, ECW, ICW, and MM did not change significantly compared to baseline.

No significant differences between the study and control groups regarding mean weight, height, and BMI were found, nor when individual results were analyzed in relation to Polish normative values.

## 4. Discussion

The primary objective of our study was to assess PA values in children and adolescents with newly diagnosed type 1 diabetes in comparison to healthy subjects, which has not yet been studied. The PA value is calculated from resistance (R) and reactance (Xc) values and reflects the nutritional and functional status. In the human body, resistance is related to tissue hydration while reactance occurs due to the cell membrane capacitance (i.e., the ability of the non-conducting object to save electrical charges) [8]. Data regarding PA assessment in the diabetic population are scarce. In the study by Buscemi et al. conducted in both type 1 and type 2 diabetic subjects, PA values appeared to be significantly lower in young adult type 1 diabetic male patients compared to control subjects, while in women, this difference was insignificant [16]. Dittmar et al. revealed significantly lower PA among men and women with type 2 diabetes compared to control subjects. They also found inverse relationships between the PA value and HbA1c levels and concluded that lower PA values at 50 and 100 kHz might indicate catabolism and long duration of the disease in type 2 diabetic patients [17]. No other studies assessing PA values in patients with diabetes were found in the PubMed, Scopus, or EMBASE databases.

Obviously, body composition of children and adolescents with type 1 diabetes significantly changes over the course of the disease, and, in many of them, an excess of fat mass develops, which was observed in different studies. Davis et al. observed a significantly lower FM% and similar lean mass in children with type 1 diabetes at the time of diagnosis compared to the non-diabetic group. After insulin introduction, sharp increases in fat mass and an insignificant loss of lean body mass during the first six weeks of treatment in diabetic group was revealed. The authors explained this phenomenon as a consequence of severe insulin deficiency and a catabolic state at the time of type 1 diabetes diagnosis [22]. In contrast to this study, in our observations, neither fat mass nor percentage of body fat in children and adolescents with newly diagnosed type 1 diabetes were significantly different compared to the control group, though we found a significantly lower percentage of muscle mass. We did not observe significant changes in body composition during the follow-up in the study group; however, the follow-up period was not long enough to find more pronounced changes. Interestingly, in the Greek study in children and adolescents with short-lasting type 1 diabetes (3.7 ± 2.0 years), FFMI was significantly higher compared to non-diabetic subjects, while FM% and FMI were not significantly different between the groups [23]. Patients with type 1 diabetes gain more weight than their peers, mainly due to the increase in fat mass, which was documented in the study by Szadkowska et al. In their observation, the prevalence of overweight and obesity among young adults (up to 40 years) with type 1 diabetes and an age at diabetes onset <20 years was significantly higher compared to the control group, and diabetic patients were characterized by higher fat mass [24]. Such unfavorable changes in body composition might have a negative impact on health in the future. Excessive weight gain in diabetic patients was associated with elevated waist circumference and blood pressure, lower insulin sensitivity, dyslipidemia, and with more extensive atherosclerosis in further observations [25,26].

It is well known that type 1 diabetes is associated with an elevated risk of complications, and poor metabolic control correlates with microangiopathy, macroangiopathy, and diabetic neuropathy [26,27]. Uncontrolled type 1 diabetes also has an impact on bone and muscle, i.e., lean body mass; moreover, it also impairs their function [28,29]. Coleman et al. indicate that decreased muscle mass and impaired muscle function has a significant impact on insulin sensitivity, glucose and lipids disposal, and on basal metabolic rate, which makes it difficult to achieve a stable metabolic control of type 1 diabetes. Thus, physical activity, which leads to an improvement in muscle mass and function, can also be helpful in the improvement of metabolic control and in delaying or even preventing the development of diabetic complications [30].

Poor metabolic control in childhood and adolescence and bad glycemic legacy (metabolic memory) is associated with an elevated risk of future complications in adulthood and can also lead to premature death, mainly due to cardiovascular disease. Thus, good diabetes care in early years can improve this prognosis [26,31].

Phase angle has not yet been analyzed prospectively in type 1 diabetic patients. The significant increases in PA values during the follow-up observed in our study can be simply explained by the growth of children and adolescents, because number of subjects with PA exceeding mean for age, gender, and BMI did not increase; on the contrary, it even decreased during observation. This may indicate the persistence of worse nutritional and functional status in this population.

The main limitation of our study was the relatively small number of participants, which did not allow us to find other differences between diabetic and control groups. Furthermore, only thirteen patients with diabetes were followed-up. Nevertheless, our study presents some new data and interesting findings which may indicate the directions of future research.

## 5. Conclusions

Low values of phase angle and altered body composition in newly diagnosed type 1 diabetic patients indicate their worse nutritional and functional status. However, although phase angle is a predictor of unfavorable clinical outcome in people with a number of chronic diseases, the predictive and prognostic value of our findings in patients with type 1 diabetes needs to be determined in further long-term prospective studies to establish its role in this population.

## Figures and Tables

**Table 1:** Demographic and anthropometric parameters of the study participants.

Parameter	Type 1 Diabetes Group	Control Group	*p*-Value
Age (years)	10.78 ± 3.72	10.80 ± 3.73	0.967
Girls	10.93 ± 3.55	10.96 ± 3.56	0.947
Boys	10.66 ± 3.90	10.66 ± 3.90	1.000
Body weight (kg)	42.15 ± 21.08	39.93 ± 14.97	0.907
Girls	38.71 ± 16.69	38.53 ± 11.92	0.962
Percentile * (*n*):			
<3	2	1	0.613
3–10	4	1	
10–90	20	23	
90–97	1	1	
>97	1	2	
Boys	44.89 ± 23.91	41.05 ± 17.11	0.720
Percentile * (*n*):			
<3	0	1	0.400
3–10	4	2	
10–90	24	26	
90–97	3	5	
>97	4	1	
Height (cm)	145.80 ± 22.16	144.12 ± 19.63	0.653
Girls	143.93 ± 19.64	143.66 ± 18.60	0.958
Low-to-age *	1	1	0.354
Normal *	27	25	
High-to-age *	0	2	
Boys	147.30 ± 24.17	144.49 ± 20.68	0.602
Low-to-age *	0	0	0.673
Normal *	31	33	
High-to-age *	4	2	
BMI (kg/m^2^)	18.56 ± 4.57	18.52 ± 3.30	0.460
Girls	17.76 ± 3.40	18.26 ± 3.08	0.244
Underweight *	10	5	0.371
Normal weight *	15	19	
Overweight *	3	3	
Obesity *	0	1	
Boys	19.21 ± 4.94	18.72 ± 3.49	0.939
Underweight *	1	1	0.573
Normal weight *	27	24	
Overweight *	6	10	
Obesity *	1	0	

SD—standard deviation; BMI—body mass index. Values are presented as mean ± SD. * According to Polish normative values [18].

**Table 2:** Bioelectrical Impedance Analysis results in study participants.

Parameter	Type 1 Diabetes Group (*N* = 63)	Control group (*N* = 63)	*p*-Value
	Mean ± SD	Mean ± SD	
Fat mass (kg)	10.84 ± 8.33	9.07 ± 5.20	0.481
Fat mass (% of body mass)	23.96 ± 9.50	22.49 ± 9.44	0.386
Fat mass index (kg/m^2^)	4.66 ± 2.78	4.24 ± 2.33	0.364
Fat free mass (kg)	31.31 ± 14.49	30.86 ± 12.26	0.840
Fat free mass (% of body mass)	76.04 ± 9.50	77.51 ± 9.44	0.386
Fat free mass index (kg/m^2^)	13.81 ± 2.74	14.20 ± 2.26	0.218
Muscle mass (kg)	18.87 ± 10.01	20.03 ± 9.17	0.341
Muscle mass (% of body mass)	44.61 ± 6.58	49.40 ± 7.59	<0.001
Total body water (L)	24.48 ± 10.61	24.28 ± 9.10	0.903
Total body water (% of body mass)	59.95 ± 8.62	61.36 ± 8.56	0.359
Extracellular water (L)	11.10 ± 4.44	10.57 ± 3.85	0.707
Extracellular water (% of body mass)	46.89 ± 7.34	44.05 ± 3.67	0.064
Intracellular water (L)	13.70 ± 6.49	13.76 ± 5.44	0.946
Intracellular water (% of body mass)	53.11 ± 7.34	55.95 ± 3.67	0.064
Body cell mass (kg)	15.14 ± 8.27	16.23 ± 7.57	0.308
Body cell mass (% of body mass)	46.89 ± 5.67	51.40 ± 4.19	<0.001
Body cell mass index (kg/m^2^)	6.57 ± 1.80	7.37 ± 1.72	0.004
Resistance (ohm)	684.92 ± 99.29	659.98 ± 94.10	0.150
Reactance (ohm)	57.62 ± 10.41	63.83 ± 6.93	<0.001
Phase angle (−)	4.85 ± 0.86	5.62 ± 0.81	<0.001

A tetrapolar system with a contralateral mode; amplitude of a measured current of 800 uA, sinusoidal, 50kHz. Significant differences are in bold and indicate significant values (*p* < 0.05). Values are presented as mean ± SD.

**Table 3:** BIA results in study participants according to gender.

Parameter	Girls		*p*-Value	Boys		*p*-Value
	Diabetes (*N* = 28)	Control (*N* = 28)		Diabetes (*N* = 35)	Control (*N* = 35)	
Fat mass (kg)	9.98 ± 6.53	9.77 ± 4.53	0.891	11.53 ± 9.57	8.50 ± 5.67	0.215
Fat mass (% of body mass)	23.65 ± 9.72	25.12 ± 8.56	0.555	24.21 ± 9.46	20.38 ± 9.47	0.072
Fat mass index (kg/m^2^)	4.37 ± 2.40	4.71 ± 2.39	0.611	4.90 ± 3.06	3.86 ± 2.25	0.079
Fat free mass (kg)	28.74 ± 11.00	28.76 ± 9.52	0.994	33.37 ± 16.63	32.55 ± 13.98	0.930
Fat free mass (% of body mass)	76.35 ± 9.72	74.88 ± 8.86	0.555	75.79 ± 9.46	79.62 ± 9.47	0.072
Fat free mass index (kg/m^2^)	13.18 ± 2.52	13.46 ± 1.78	0.628	14.32 ± 2.84	14.78 ± 2.45	0.247
Muscle mass (kg)	16.79 ± 7.25	18.29 ± 6.59	0.421	20.53 ± 11.60	21.42 ± 10.70	0.597
Muscle mass (% of body mass)	43.62 ± 5.70	47.20 ± 6.17	0.028	45.41 ± 7.19	51.15 ± 8.23	0.003
Total body water (L)	22.25 ± 7.82	22.35 ± 6.92	0.958	26.25 ± 12.23	25.81 ± 10.37	0.893
Total body water (% of body mass)	59.75 ± 9.02	58.55 ± 7.54	0.590	60.11 ± 8.42	63.61 ± 8.77	0.093
Extracellular water (L)	10.48 ± 3.64	9.85 ± 3.12	0.629	11.59 ± 4.98	11.15 ± 4.30	0.930
Extracellular water (% of body mass)	48.31 ± 8.70	44.25 ± 3.08	0.119	45.76 ± 5.92	43.89 ± 4.12	0.131
Intracellular water (L)	12.13 ± 4.99	12.65 ± 4.05	0.793	14.95 ± 7.31	14.65 ± 6.25	0.995
Intracellular water (% of body mass)	51.69 ± 8.70	55.75 ± 3.08	0.119	54.24 ± 5.92	56.11 ± 4.12	0.131
Body cell mass (kg)	13.39 ± 5.99	14.79 ± 5.38	0.362	16.55 ± 9.57	17.37 ± 8.86	0.565
Body cell mass (% of body mass)	45.60 ± 6.30	50.90 ± 3.05	<0.001	47.91 ± 4.96	51.79 ± 4.93	0.002
Body cell mass index (kg/m^2^)	6.11 ± 1.50	6.91 ± 1.27	0.037	6.93 ± 1.95	7.73 ± 1.96	0.039
Resistance (ohm)	712.75 ± 102.10	700.57 ± 79.01	0.620	662.66 ± 92.47	627.51 ± 93.53	0.119
Reactance (ohm)	58.07 ± 12.66	66.71 ± 5.14	0.003	57.26 ± 8.38	61.51 ± 7.36	0.027
Phase angle (−)	4.66 ± 0.84	5.49 ± 0.56	<0.001	5.00 ± 0.85	5.72 ± 0.97	0.001

A tetrapolar system with a contralateral mode; amplitude of a measured current of 800 uA, sinusoidal, 50kHz. Significant differences (*p* < 0.05) indicated in bold. Values are presented as mean ± SD.

**Table 4:** Number of subjects with PA value according to age, gender and BMI in relation to reference values.

Age Range (years)	Phase Angle Percentiles	Girls		Boys	
		Diabetes	Control	Diabetes	Control
All (*n*)		28	28	35	35
	≥10th percentile	14	27	20	30
	<10th percentile	14	1	15	5
	<5th percentile	13	1	11	1
4–9 (*n*)		10	10	13	13
	≥10th percentile	3	10	7	10
	<10th percentile	7	0	6	3
	<5th percentile	7	0	5	0
10–13 (*n*)		13	13	16	16
	≥10th percentile	9	13	10	14
	<10th percentile	4	0	6	2
	<5th percentile	3	0	5	1
14–18 (*n*)		5	5	6	6
	≥10th percentile	2	4	3	6
	<10th percentile	3	1	3	0
	<5th percentile	3	1	1	0

Phase angle (PA) value ≥10th percentile, <10th percentile and <5th percentile according to age, gender and body mass index (BMI) in relation to reference values from Bosy-Westphal et al. [9].
